# Dietary B Vitamins and a 10-Year Risk of Dementia in Older Persons

**DOI:** 10.3390/nu8120761

**Published:** 2016-11-26

**Authors:** Sophie Lefèvre-Arbogast, Catherine Féart, Jean-François Dartigues, Catherine Helmer, Luc Letenneur, Cécilia Samieri

**Affiliations:** 1 University Bordeaux, Institut de Santé Publique d’Epidémiologie et de Développement (ISPED), Centre INSERM U1219-Bordeaux Population Health, Bordeaux 33076, France; Catherine.Feart@isped.u-bordeaux2.fr (C.F.); Jean-Francois.Dartigues@isped.u-bordeaux2.fr (J.-F.D.); Catherine.Helmer@isped.u-bordeaux2.fr (C.H.); luc.letenneur@inserm.fr (L.L.); Cecilia.Samieri@isped.u-bordeaux2.fr (C.S.); 2 Institut National de la Santé et de la Recherche Médicale (INSERM), Institut de Santé Publique d’Epidémiologie et de Développement (ISPED), Centre INSERM U1219-Bordeaux Population Health, Bordeaux 33076, France

**Keywords:** folate, B vitamins, dementia, aging

## Abstract

B vitamins may lower the risk of dementia, yet epidemiological findings, mostly from countries with folic acid fortification, have remained inconsistent. We evaluated in a large French cohort of older persons the associations between dietary B vitamins and long-term incident dementia. We included 1321 participants from the Three-City Study who completed a 24 h dietary recall, were free of dementia at the time of diet assessment, and were followed for an average of 7.4 years. In Cox proportional hazards models adjusted for multiple potential confounders, including overall diet quality, higher intake of folate was inversely associated with the risk of dementia (*p* for trend = 0.02), with an approximately 50% lower risk for individuals in the highest compared to the lowest quintile of folate (HR = 0.47; 95% CI 0.28; 0.81). No association was found for vitamins B6 and B12. In conclusion, in a large French cohort with a relatively low baseline folate status (average intake = 278 µg/day), higher folate intakes were associated with a decreased risk of dementia.

## 1. Introduction

B vitamins have fundamental roles in the function of the central nervous system [1], and have been considered as promising candidates for the prevention of dementia and its major etiology, Alzheimer’s disease (AD). B vitamins (B6, folate, and B12) regulate homocysteine (Hcy) levels, and hyperhomocysteinemia is a major vascular risk factor and an established risk factor for dementia [2]. Hyperhomocysteinemia may affect brain function through both vascular mediation and neurotoxicity [3]. B vitamins may also have a direct effect on the brain, independently of homocysteine [4]. Indeed, a lower status in B vitamins decreases *S*-adenosylmethionine, a major intermediate of methylation reactions in the brain which are involved in synaptic transmission and epigenetic regulation [5]. Moreover, tetrahydrofolate, the active form of folate, is involved in DNA repair system [6] and DNA replication, which are both critical for adult hippocampal neurogenesis [7]. Direct mechanistic effects of B vitamins on the brain independent of Hcy may therefore be particularly relevant to folate [8,9,10].

Prospective epidemiological studies have found associations between higher intakes of B vitamins and a lower risk of dementia and AD; three studies have reported associations with dietary folate (but not with vitamins B6 and B12) [11,12,13], although inconsistent findings have also been found, with studies reporting null [14,15] or even harmful associations between folate intakes at high levels and cognitive function, when combined with low vitamin B12 status [16]. Findings have also been inconsistent in studies using blood status in B vitamins (see [17] for a review). Moreover, prevention trials have been inconclusive to date [18], with a few exceptions, including the two European FACIT and VITACOG trials, which evidenced the beneficial effect of folic acid on cognitive function and brain atrophy, respectively [19,20]. Although the reasons for such inconsistencies remain unclear, it is possible that the findings of some studies have been influenced by the folic acid fortification of all flour and cereal-grain products that became mandatory starting in 1998 in the US and Canada, leading to a subsequent dramatic increase in folate status in North American populations [21]. The vast majority of observational studies on B vitamins and cognitive aging have been conducted in the US, and limited research has examined European populations. In the present study, we evaluated in a large cohort of older persons from France—a country not subject to folic acid fortification policy—the association between dietary B vitamins and the risk of dementia and AD over 10 years of follow-up.

## 2. Materials and Methods

### 2.1. Study Population

Our analysis was based on the Three-City (3C) study, a French population-based cohort on dementia which started in 1999–2000, including 9294 non-institutionalized community dwellers aged 65 years or older in three French cities: Bordeaux (*n* = 2104), Dijon (*n* = 4931), and Montpellier (*n* = 2259). Face-to-face interviews were performed at the cohort baseline with the collection of sociodemographic, lifestyle, and health-related characteristics, neuropsychological testing, and blood sampling. The protocol of the 3C study has been described in detail previously [22]. The Consultative Committee for the Protection of Persons participating in Biomedical Research at Kremlin-Bicêtre University Hospital (Paris, France) approved the 3C study protocol (Project no. 99-28, june 1999) and all participants provided written informed consent. Follow-up examinations were conducted 2 years (2001–2002), 4 years (2003–2004), 7 years (2006–2007), 10 years (2009–2010), and 12 years (2011–2012) after the cohort baseline. The present study is based on data from Bordeaux, the only 3C center with a dietary assessment.

**Figure 1:**
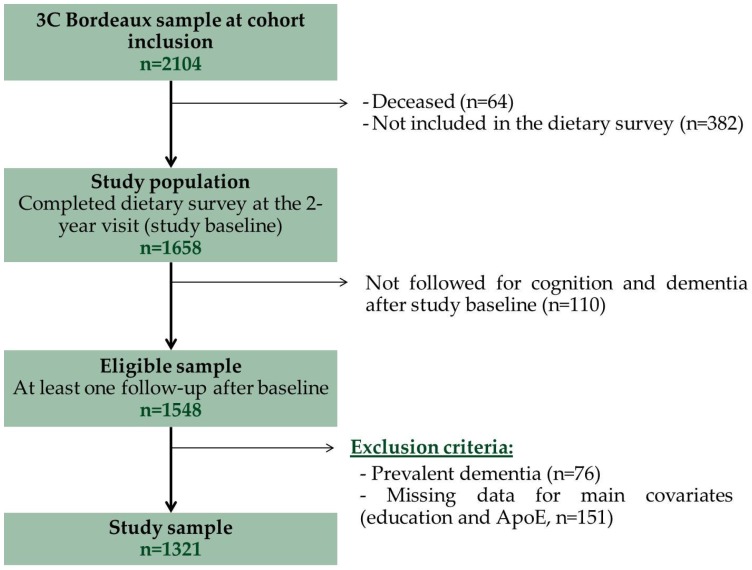
Flow diagram of the study sample selection, the 3C Bordeaux cohort. Abbreviations: 3C: Three-City; ApoE: apolipoprotein genotype.

At the 2-year visit in 2001–2002, a comprehensive dietary survey was conducted by trained dieticians, including a food frequency questionnaire (FFQ) and a 24 h dietary recall (which served as baseline in the present analysis). Figure 1 describes the procedure for the study sample selection. Our final study population included 1321 participants followed for 10 years after the dietary assessment.

### 2.2. Diagnosis of Dementia

Incident dementia was ascertained through a three-stage procedure. First, participants underwent a battery of neuropsychological tests at home conducted by a trained psychologist. Individuals suspected of dementia based on their neuropsychological performances were secondarily examined by a neurologist to establish a clinical diagnosis. Finally, all potential cases of dementia were reviewed by an independent committee of neurologists to set up consensus on dementia diagnosis and etiology, according to the *Diagnostic and Statistical Manual of Mental Disorders* (Fourth Edition) [23] and the *National Institute of Neurological and Communicative Disorders and Stroke and the Alzheimer’s Disease and Related Disorders Association* Alzheimer’s criteria [24].

### 2.3. Assessment of Diet and Intake of B-Vitamins

The procedure of the 24 h recall data collection was detailed in a previous publication [25]. Briefly, the 24 h dietary recall consisted in reporting all meals and beverages consumed the day prior to the interview (excluding week-end meals). For each food reported, portion sizes were registered using a book of photographs [26] and were used to estimate the quantity (in grams per day) of intake of each food item. Nutrient and total energy intakes were then estimated using several French food composition tables [27,28,29]. The contribution of supplemental folic acid (from fortified foods or supplements) to total folate intake is very low and virtually all folic acid is of natural origin in French populations [30]. In our analyses, we focused on intakes of B vitamins involved in one-carbon metabolism, i.e., vitamin B6, folate, and vitamin B12.

### 2.4. Other Variables

Sociodemographic information included age at study baseline, gender, and level of education. ApoEε4 genotype was defined as carrying at least one ε4 allele vs. no ε4 allele. Lifestyle factors included alcohol consumption, tobacco use, and regular exercise. To control for overall diet quality, we also computed a score of adherence to the Mediterranean diet (MeDi); the methodology for computation of the MeDi score has been described in previous publications [31,32]. Cardiovascular risk factors included body mass index (BMI), hypercholesterolemia (total cholesterol ≥6.2 mmol/L or anti-cholesterol medication), diabetes (fasting glucose ≥7.2 mmol/L or antidiabetic medication), history of cardiovascular diseases (including myocardial infarction and stroke), and hypertension (measured blood pressure higher than 140/90 mm Hg or antihypertensive medication). Depressive symptomatology was assessed using the Center for Epidemiological Studies-Depression (CES-D) [33] Scale; we defined high depressive symptoms as having a CES-D score higher than 17 in men and 23 in women (over a maximum of 60 [34]) or when the participant was “too depressed to answer”. The number of drugs regularly consumed was recorded and used as a general indicator of comorbidities.

### 2.5. Statistical Analyses

We used Cox proportional hazards models with delayed entry using age as time-scale [35] to evaluate the multivariate associations between intake of B vitamins and the risk of all-cause dementia over 10 years; we secondarily examined AD specifically. Vitamin B6, folate, and vitamin B12 were modeled simultaneously and considered in quintiles. We first adjusted for the main risk factors of dementia (gender, education, and ApoE genotype), energy intake, and season of the 24 h recall (Model 1). We further controlled for lifestyle factors, cardiovascular risk factors, and other comorbidities (Model 2).

We examined linear trends across quintiles of B vitamins by using a continuous variable taking the median intake value of each quintile. Moreover, we investigated potential interactions of biological relevance between (i) folate and alcohol intakes [36]; (ii) folate and vitamin B12 intakes [37,38]; (iii) B vitamins and long-chain omega-3 fatty acid intakes [39]; and (iv) B vitamin intakes and ApoEε4 genotype. Missing data were treated as follows. For most covariates, data were missing for less than 3% of the sample; we thus assigned to missing data the reference category (for categorical variables) or the median value (for continuous variables). Regular exercise was missing for 11.8% of the sample; we thus created a specific missing category for this variable. The proportional hazard assumption was controlled, as well as the log-linearity assumption for continuous covariates.

In sensitivity analyses, we assessed the robustness of our findings by (i) excluding participants with the highest values for vitamin B intakes, i.e., with intakes >95th percentile of at least one of the 3 B vitamins; (ii) adjusting for regular intake of fruits and vegetables instead of the Mediterranean diet score; and (iii) controlling for other potential confounders with very low prevalence, i.e., consumption of B vitamin supplements. We also controlled for potential reverse causation (which occurs when subtle cognitive impairment modifies dietary intakes) by adjusting our multivariate models for global cognitive performances at baseline, represented by the Mini Mental State Examination score [40].

Statistical analyses were performed using SAS software version 9.3 (SAS Institute Inc., Cary, NC, USA) and R software version 3.2.3 (R Foundation for Statistical Computing, Vienna, Austria).

## 3. Results

### 3.1. Characteristics of the Sample

Study participants were 75.8 years old on average (range: 67.7–94.9) at the time of the dietary survey and 62.2% were female (Table 1). Nearly 41% of participants had reached high school level or above, and 18% were ApoEε4 carriers; baseline characteristics including lifestyle and cardiovascular risk factors have been provided in Table 1. A total of 197 individuals (15% of the sample) developed dementia over 7.4 years of follow-up on average (range: 0.9–11.2), including 131 classified with possible or probable AD (351 participants died during follow-up and 192 refused or were lost to follow-up). The incidence rate of dementia was 2.0 cases per 100 person-years, and the incidence of AD was 1.3 per 100 person-years.

Mean daily intakes were 1.5 mg/day for vitamin B6, 278.3 µg/day for folate, and 5.7 µg/day for vitamin B12 (Table 1). Compared with individuals who remained free of dementia during follow-up, those who developed the disease had lower B vitamin intakes at baseline, although the difference was statistically significant for folate only in univariate Cox models (*p* = 0.01 for risk of dementia for each 1SD-increase of folate intake), without any marked difference in daily energy intake across both groups (*p* = 0.44).

### 3.2. Multivariate Associations between Intake of B Vitamins and Risk of Dementia

In multivariate analyses, a higher intake of folate was significantly associated with a lower risk of dementia (*p* for trend ≤ 0.02 across increasing quintiles in multivariate models, Table 2). Adjustments for potential confounders did not markedly alter HRs, suggesting a minimal confounding effect. After controlling for a large set of potential confounders in Model 2, compared with individuals in the lowest quintile of folate, those in the highest quintile had a 47% lower risk of dementia over 10 years (HR (95% CI) = 0.47 (0.28, 0.81)). In contrast, we did not find any significant association between vitamin B6 or vitamin B12 intakes and the risk of dementia (*p* for trend = 0.38 and 0.73, respectively, in Model 2).

When we examined the risk of AD, we found similar associations of high folate with a lower risk of AD, although the magnitude of associations was slightly weaker than those found with all-cause dementia (*p* for trend = 0.08 in the first model, HR for higher versus lower quintile of folate = 0.52, 95% CI = 0.28, 0.97; results are not shown in the tables) and relationships were attenuated and no longer statistically significant after further adjustments (*p* for trend = 0.17 in Model 2).

We did not detect any significant interaction between B vitamins and alcohol, and omega-3 intakes or with ApoEε4 carrier status. Likewise, there was no significant interaction between folate and vitamin B12 intakes on dementia risk.

### 3.3. Secondary Analyses

When we conducted sensitivity analysis by further adjusting models for baseline cognition, our results remained unchanged, suggesting that findings may not be due to reverse causation by incipient prodromal dementia. Likewise, a sensitivity analysis excluding the *n* = 155 participants with higher intake values for at least one of the 3 B vitamins did not modify results, suggesting that findings were not driven by a few particular individuals with extreme intake values. Results were also virtually unchanged when adjusting for regular fruit and vegetable consumption instead of the MeDi score, suggesting minimal confounding by other potentially beneficial nutrients contained in the major food source of dietary folate. Very few people (*n* = 19) consumed B vitamin/multivitamin supplements in this French population, and the inclusion of B vitamin/multivitamin supplementation in our models did not modify findings.

## 4. Discussion

In this large French cohort of older persons followed for more than a decade, we found that higher folate intakes were inversely associated with the risk of dementia, independently of intakes of other B vitamins, overall diet quality, and a large set of other potential confounders. Compared with individuals with folate intakes in the lowest quintile, those in the higher quintile had an approximately 50% lower risk of dementia. A similar trend was found with AD risk, although associations were weaker and no longer significant after adjustment for the full set of potential confounders (possibly due to less power to detect a significant relationship with AD cases, which represented only 66% of all-cause dementia cases). In contrast to folate, there was no significant association between dietary vitamins B6 and B12 and the risk of dementia or AD in this large cohort.

Our results are consistent with at least three previous US longitudinal studies, in which higher folate intake was associated with a lower risk of dementia or AD, while associations of dietary vitamins B6 and B12 to dementia risk were not significant [11,12,13]. In contrast, our results are not consistent with two other previous large US cohorts, the Chicago Health and Aging Project (CHAP) and the Cache County Memory Study (CCMS), which did not evidence any association between quintiles of B vitamin intake and risk of AD [14,15]; harmful associations between higher folate and cognition have also been found among persons with low vitamin B12 status in several cohorts [16,37,38]. The reasons for inconsistencies between studies are unclear. The most evident reason may pertain to differences in baseline intakes between French and US populations, with lower intakes reported in France, a country with limited supplement use and no folic acid fortification (e.g., median total folate intake in the upper quintile was 444 µg/day in our 3C sample, compared to 742 µg/day in the CHAP study [16]). It is thus possible that folate is protective for the brain in lower intake ranges (as those observed in France) and becomes inefficient—and even detrimental for those with low B12 status—at higher ranges. Otherwise, findings of US cohorts have remained difficult to interpret due to the fortification policy introduced in the late 1990s. Indeed, part of the dietary data in the CHAP study was collected after folic acid fortification; furthermore, although diet was ascertained in 1995 (i.e., pre-folic acid fortification) in the CCMS, follow-up for cognition and dementia was conducted through 2004, with >70% of the follow-up period for cognition covering post-folic acid fortification. It is thus possible that the increase in folate status in the US population which occurred after folic acid fortification have biased these studies towards the null.

Additionally, although most trials failed to evidence any benefit of B vitamin supplementation on cognitive function, our results are nonetheless consistent with three European trials which found a protective effect of B vitamins on brain aging outcomes among persons with raised Hcy or with low folate status [19,20,41,42,43]. In Italy, a small pilot trial found a benefit of folic acid supplementation on memory and attention among older persons selected with low initial status in folate [43]. In The Netherlands, the large FACIT trial included participants aged ≥50 years old with high levels of Hcy and found a significant benefit of folic acid supplementation over three years on memory and processing speed [19]; in the UK, the VITACOG study included older persons aged ≥70 years old with mild cognitive impairment (without a priori selection based on Hcy levels) and found an efficacy of B vitamins on brain atrophy [20,41], and on cognition limited to those with raised Hcy at baseline [42]. Finally, in the single largest French study to date based on secondary analyses on cognition from the large SU.FOL.OM3 trial on B vitamins and omega-3 fatty acids for secondary prevention of cardiovascular diseases, a protective effect of B vitamins was evidenced on global cognition and memory in the older age group (65–80 years) [44]. Together with our own study, these results suggest a beneficial effect of B vitamins on cognition in older persons (especially among those with raised Hcy or low folate status).

In contrast to folate, dietary vitamins B6 and B12 were not significantly associated with the risk of dementia or AD in the present study. Although several longitudinal studies found inverse associations between blood markers of vitamin B12 and brain health [45,46,47,48,49] (associations potentially fully mediated by Hcy [47,48]), studies generally found, as in our study, null results when examining dietary B6 and B12 and cognitive health [11,12,13,14,15,16]. Indeed, studies based on intake data (including our own study) may not capture food-bound vitamin B12 malabsorption, which is common in older persons and adversely affects vitamin B12 status in healthy older people irrespective of dietary intakes [50]. Thus, circulating vitamin B12 may be a better marker of vitamin B12 deficiency than vitamin B12 intakes in older populations.

A limitation of the present study is that a single 24 h recall was used to assess B vitamin intake. A reported single day of intake does not fully capture intra-individual variation in dietary intakes, and might lead to misclassification, especially for foods consumed occasionally (such as offal, a top source for vitamin B12). However, in large samples, a single 24 h provides acceptable estimations of average intakes in subgroups of a population [51]; accordingly, we observed in our study average intake values similar to other studies in older populations for macronutrients [25] and for micronutrients including B vitamins (e.g., average intake values for B vitamins in our sample were close to those observed among older persons from the French Individual and National Study on Food Consumption (INCA 2) [52]—a national representative survey). In addition, the use of dietary supplements may not have been accurately recorded by our questionnaire [53]. Moreover, as in any observational study, there might be residual confounding. In particular, B vitamin intake may reflect an overall healthy diet, and we did not adjust for other healthy nutrients which may exert protective effects to the brain. However, we were able to control for a well-established index of overall diet quality, the MeDi score, and further adjustment for the MeDi revealed minimal confounding by global diet quality.

Our study also has important strengths. Obvious strengths include a longitudinal design with a decade of follow-up, and a multi-step procedure of diagnosis of dementia including a validation of cases by an independent committee of neurologists. The long lag period between the ascertainment of B vitamin intakes and the diagnosis of dementia minimizes the possibility of reverse causation. Finally, we considered a large range of potential confounders, including cardiovascular and lifestyle factors, such as the overall diet quality, and adjusting for these factors did not markedly decrease our risk estimate, suggesting minimal confounding effect.

## 5. Conclusions

In conclusion, we found in a large cohort of older persons from France—a country with no folic acid fortification and relatively low average intake levels—a strong association between a higher intake of folate and a lower long-term risk of dementia, while there was no evidence of association of vitamin B6 and B12 intake to dementia risk; however, vitamin B12 malabsorption has not been explored through circulating level data in this cohort of older persons and deserves further research. The protective role of folate in populations with relatively low basal folate status such as France may be worth exploring in future dementia prevention trials.

## Figures and Tables

**Table 1:** Baseline characteristics and B vitamin intakes of the participants according to incident all-cause dementia over 10 years, the 3C Bordeaux cohort (*n* = 1321).

Baseline Characteristics	Overall Sample (*n* = 1321)	Incident Dementia (*n* = 197)	No Dementia (*n* = 1124)	*p* for Risk of Dementia ^a^
Age (years)	75.8 ± 4.8	78.3 ± 4.6	75.4 ± 4.7	<0.001
Gender, female	822 (62.2)	140 (71.1)	682 (60.7)	0.30
Education ≥high school	540 (40.9)	72 (36.5)	468 (41.6)	0.24
ApoEε4, carrier	240 (18.2)	49 (24.9)	191 (17.0)	<0.001
Alcohol intake (g/day)	13.9 ± 15.5	12.9 ± 14.0	14.1 ± 15.8	0.86
Tobacco consumption (pack-year)				
0	861 (65.2)	142 (72.1)	719 (64.0)	0.75
<10	156 (11.8)	20 (10.2)	136 (12.1)	
(10–20)	84 (6.4)	10 (5.1)	74 (6.6)	
(20–30)	71 (5.4)	7 (3.6)	64 (5.7)	
≥30	149 (11.3)	18 (9.1)	131 (11.7)	
Regular exercise ^b^	412 (35.3)	46 (28.2)	366 (36.5)	0.14
BMI (kg/m^2^)	26.6 ± 4.1	26.1 ± 4.3	26.7 ± 4.1	0.23
Hypercholesterolemia	762 (57.7)	125 (63.5)	637 (56.7)	0.16
Diabetes	125 (9.5)	33 (16.8)	92 (8.2)	<0.001
History of cardiovascular diseases	428 (32.4)	63 (32.0)	365 (32.5)	0.94
Hypertension	998 (75.5)	151 (76.6)	847 (75.4)	0.63
High depressive symptomatology	98 (7.4)	22 (11.2)	76 (6.8)	0.10
Number of drugs consumed	4.8 ± 2.9	5.8 ± 3.2	4.6 ± 2.8	<0.001
B vitamin/multivitamin supplement use	19 (1.4)	2 (1.0)	17 (1.5)	0.59
Energy intake (Kcal/day)	1623.0 ± 514.0	1565.0 ± 500.2	1633.0 ± 515.9	0.44
Vitamin B6 intake (mg/day)	1.5 ± 0.6	1.4 ± 0.6	1.5 ± 0.6	0.52
Folate intake (µg/day)	278.3 ± 134.8	251.9 ± 126.1	283.0 ± 135.8	0.01
Vitamin B12 intake (µg/day)	5.7 ± 11.4	4.9 ± 9.9	5.8 ± 11.7	0.30

Values are mean ± SD or number (percentages). ^a^ Univariate Cox Proportional Hazards models with delayed entry using age as time scale, except for age which use standard Cox Proportional Hazards models; ^b^ Percentages are of non-missing values. Abbreviations: ApoEε4: allele ε4 for the apolipoprotein E gene; BMI: body mass index.

**Table 2:** Multivariate associations between quintiles of B vitamin intake and risk of all-cause dementia over 10 years, the 3C Bordeaux cohort (*n* = 1321).

	Number of Dementia Cases	Risk of Dementia (HR [95% CI]) ^a^	
		Model 1	Model 2
**Vitamin B6 (mg/day)**			
Q1 <1.0	50	1.0 (reference)	1.0 (reference)
Q2 (1.0–1.2)	36	0.81 (0.51–1.28)	0.86 (0.54–1.36)
Q3 (1.2–1.5)	36	1.02 (0.63–1.65)	1.08 (0.66–1.77)
Q4 (1.5–1.9)	42	1.26 (0.78–2.04)	1.40 (0.85–2.31)
Q5 ≥1.9	33	1.02 (0.58–1.78)	1.08 (0.60–1.94)
*p* for trend		0.52	0.38
**Folate (µg/day)**			
Q1 <168.3	56	1.0 (reference)	1.0 (reference)
Q2 (168.3–225.4)	37	0.66 (0.43–1.02)	0.66 (0.42–1.02)
Q3 (225.4–281.4)	40	0.67 (0.43–1.03)	0.73 (0.47–1.15)
Q4 (281.4–375.6)	35	0.69 (0.43–1.10)	0.76 (0.47–1.24)
Q5 ≥375.6	29	0.47 (0.28–0.79)	0.47 (0.28–0.81)
*p* for trend		0.01	0.02
**Vitamin B12 (µg/day)**			
Q1 <1.8	38	1.0 (reference)	1.0 (reference)
Q2 (1.8–2.6)	47	1.42 (0.91–2.21)	1.27 (0.81–1.98)
Q3 (2.6–3.7)	40	1.29 (0.81–2.07)	1.15 (0.71–1.86)
Q4 (3.7–5.7)	40	1.30 (0.81–2.10)	1.26 (0.77–2.05)
Q5 ≥5.7	32	1.17 (0.70–1.94)	1.04 (0.61–1.75)
*p* for trend		0.95	0.73

^a^ Cox proportional hazards models with delayed entry using age as time-scale. The three B vitamins were modeled simultaneously. Model 1: adjusted for gender, level of education, ApoEε4, energy intake, and season of the 24 h recall. Model 2: covariates from Model 1 plus alcohol and tobacco consumptions, regular exercise, the Mediterranean Diet score, BMI, hypercholesterolemia, diabetes, history of cardiovascular diseases, hypertension, depressive symptomatology, and number of drugs consumed.

## References

[1] Reynolds E. (2006). Vitamin B12, folic acid, and the nervous system. Lancet Neurol..

[2] Seshadri S., Beiser A., Selhub J., Jacques P.F., Rosenberg I.H., D’Agostino R.B., Wilson P.W.F., Wolf P.A. (2002). Plasma Homocysteine as a Risk Factor for Dementia and Alzheimer’s Disease. N. Engl. J. Med..

[3] Morris M.S. (2003). Homocysteine and Alzheimer’s disease. Lancet Neurol..

[4] Smith A.D., Refsum H. (2016). Homocysteine, B Vitamins, and Cognitive Impairment. Annu. Rev. Nutr..

[5] Selhub J., Troen A., Rosenberg I.H. (2010). B vitamins and the aging brain. Nutr. Rev..

[6] Araújo J.R., Martel F., Borges N., Araújo J.M., Keating E. (2015). Folates and aging: Role in mild cognitive impairment, dementia and depression. Ageing Res. Rev..

[7] Morris M.S. (2012). The Role of B Vitamins in Preventing and Treating Cognitive Impairment and Decline. Adv. Nutr. Int. Rev. J..

[8] Ravaglia G., Forti P., Maioli F., Martelli M., Servadei L., Brunetti N., Porcellini E., Licastro F. (2005). Homocysteine and folate as risk factors for dementia and Alzheimer disease. Am. J. Clin. Nutr..

[9] Ramos M.I., Allen L.H., Mungas D.M., Jagust W.J., Haan M.N., Green R., Miller J.W. (2005). Low folate status is associated with impaired cognitive function and dementia in the Sacramento Area Latino Study on Aging. Am. J. Clin. Nutr..

[10] De Lau L.M.L., Refsum H., Smith A.D., Johnston C., Breteler M.M.B. (2007). Plasma folate concentration and cognitive performance: Rotterdam Scan Study. Am. J. Clin. Nutr..

[11] Agnew-Blais J.C., Wassertheil-Smoller S., Kang J.H., Hogan P.E., Coker L.H., Snetselaar L.G., Smoller J.W. (2015). Folate, vitamin B-6, and vitamin B-12 intake and mild cognitive impairment and probable dementia in the Women’s Health Initiative Memory Study. J. Acad. Nutr. Diet..

[12] Corrada M.M., Kawas C.H., Hallfrisch J., Muller D., Brookmeyer R. (2005). Reduced risk of Alzheimer’s disease with high folate intake: The Baltimore Longitudinal Study of Aging. Alzheimers Dement. J. Alzheimers Assoc..

[13] Luchsinger J.A., Tang M.-X., Miller J., Green R., Mayeux R. (2007). Relation of higher folate intake to lower risk of Alzheimer disease in the elderly. Arch. Neurol..

[14] Nelson C., Wengreen H.J., Munger R.G., Corcoran C.D. (2009). Dietary folate, vitamin B-12, vitamin B-6 and incident Alzheimer’s disease: The cache county memory, health and aging study. J. Nutr. Health Aging.

[15] Morris M.C., Evans D.A., Schneider J.A., Tangney C.C., Bienia J.L., Aggarwal N.T. (2006). Dietary folate and vitamins B-12 and B-6 not associated with incident Alzheimer’s disease. J. Alzheimers Dis..

[16] Morris M.C., Evans D.A., Bienias J.L., Tangney C.C., Hebert L.E., Scherr P.A., Schneider J.A. (2005). Dietary folate and vitamin B12 intake and cognitive decline among community-dwelling older persons. Arch. Neurol..

[17] Hinterberger M., Fischer P. (2013). Folate and Alzheimer: When time matters. J. Neural Transm..

[18] Clarke R., Bennett D., Parish S., Lewington S., Skeaff M., Eussen S.J.P.M., Lewerin C., Stott D.J., Armitage J., Hankey G.J. (2014). B-Vitamin Treatment Trialists’ Collaboration Effects of homocysteine lowering with B vitamins on cognitive aging: Meta-analysis of 11 trials with cognitive data on 22,000 individuals. Am. J. Clin. Nutr..

[19] Durga J., van Boxtel M.P., Schouten E.G., Kok F.J., Jolles J., Katan M.B., Verhoef P. (2007). Effect of 3-year folic acid supplementation on cognitive function in older adults in the FACIT trial: A randomised, double blind, controlled trial. Lancet.

[20] Douaud G., Refsum H., de Jager C.A., Jacoby R.E., Nichols T., Smith S.M., Smith A.D. (2013). Preventing Alzheimer’s disease-related gray matter atrophy by B-vitamin treatment. Proc. Natl. Acad. Sci. USA.

[21] Jacques P.F., Selhub J., Bostom A.G., Wilson P.W.F., Rosenberg I.H. (1999). The Effect of Folic Acid Fortification on Plasma Folate and Total Homocysteine Concentrations. N. Engl. J. Med..

[22] 3C Study Group (2003). Vascular factors and risk of dementia: Design of the Three-City Study and baseline characteristics of the study population. Neuroepidemiology.

[23] American Psychiatric Association (1994). DSM-IV: Diagnostic and Statistical Manual of Mental Disorders.

[24] McKhann G., Drachman D., Folstein M., Katzman R., Price D., Stadlan E.M. (1984). Clinical diagnosis of Alzheimer’s disease: Report of the NINCDS-ADRDA Work Group under the auspices of Department of Health and Human Services Task Force on Alzheimer’s Disease. Neurology.

[25] Féart C., Jutand M.A., Larrieu S., Letenneur L., Delcourt C., Combe N., Barberger-Gateau P. (2007). Energy, macronutrient and fatty acid intake of French elderly community dwellers and association with socio-demographic characteristics: Data from the Bordeaux sample of the Three-City Study. Br. J. Nutr..

[26] Hercberg S., Deheeger M., Preziosi P. (2000). Portions Alimentaires: Manuel Photos Pour L’estimation des Quantités.

[27] Favier J.-C., Ireland-Ripert J., Toque C., Feinberg M. (1995). Répertoire Général des Aliments: Table de Composition.

[28] Souci S.W., Fachmann W., Kraut H. (2000). Food Composition and Nutrition Tables.

[29] Renaud S., Godsey F., Ortchanian E., Baudier F. (1979). Table de Composition des Aliments.

[30] Agence Nationale de Sécurité Sanitaire, de l’alimentation, de l’environnement et du travail (ANSES) (2015). AVIS Relatif à L’évaluation des Apports en Vitamines et Minéraux Issus de L’alimentation non Enrichie, de L’alimentation Enrichie et des Compléments Alimentaires dans la Population Française: Estimation des Apports Usuels, des Prévalences D’inadéquation et des Risques de Dépassement des Limites de Sécurité; Avis de l’ANSES. Saisine n°2012-SA-0142.

[31] Trichopoulou A., Costacou T., Bamia C., Trichopoulos D. (2003). Adherence to a Mediterranean Diet and Survival in a Greek Population. N. Engl. J. Med..

[32] Féart C., Samieri C., Rondeau V., Amieva H., Portet F., Dartigues J.F., Scarmeas N., Barberger-Gateau P. (2009). Adherence to a mediterranean diet, cognitive decline, and risk of dementia. JAMA.

[33] Radloff L.S. (1977). The CES-D Scale A Self-Report Depression Scale for Research in the General Population. Appl. Psychol. Meas..

[34] Fuhrer R., Rouillon F. (1989). The French version of the CES-D (Center for Epidemiologic Studies-Depression Scale). Psychiatr. Psychobiol..

[35] Lamarca R., Alonso J., Gómez G., Muñoz Á. (1998). Left-truncated Data with Age as Time Scale: An Alternative for Survival Analysis in the Elderly Population. J. Gerontol. A Biol. Sci. Med. Sci..

[36] Halsted C.H., Villanueva J.A., Devlin A.M., Chandler C.J. (2002). Metabolic Interactions of Alcohol and Folate. J. Nutr..

[37] Morris M.S., Jacques P.F., Rosenberg I.H., Selhub J. (2007). Folate and vitamin B-12 status in relation to anemia, macrocytosis, and cognitive impairment in older Americans in the age of folic acid fortification. Am. J. Clin. Nutr..

[38] Moore E.M., Ames D., Mander A.G., Carne R.P., Brodaty H., Woodward M.C., Boundy K., Ellis K.A., Bush A.I., Faux N.G. (2014). Among vitamin B12 deficient older people, high folate levels are associated with worse cognitive function: Combined data from three cohorts. J. Alzheimers Dis..

[39] Jernerén F., Elshorbagy A.K., Oulhaj A., Smith S.M., Refsum H., Smith A.D. (2015). Brain atrophy in cognitively impaired elderly: The importance of long-chain ω-3 fatty acids and B vitamin status in a randomized controlled trial. Am. J. Clin. Nutr..

[40] Folstein M.F., Folstein S.E., McHugh P.R. (1975). “Mini-mental state”: A practical method for grading the cognitive state of patients for the clinician. J. Psychiatr. Res..

[41] Smith A.D., Smith S.M., de Jager C.A., Whitbread P., Johnston C., Agacinski G., Oulhaj A., Bradley K.M., Jacoby R., Refsum H. (2010). Homocysteine-lowering by B vitamins slows the rate of accelerated brain atrophy in mild cognitive impairment: A randomized controlled trial. PLoS ONE.

[42] De Jager C.A., Oulhaj A., Jacoby R., Refsum H., Smith A.D. (2012). Cognitive and clinical outcomes of homocysteine-lowering B-vitamin treatment in mild cognitive impairment: A randomized controlled trial. Int. J. Geriatr. Psychiatry.

[43] Fioravanti M., Ferrario E., Massaia M., Cappa G., Rivolta G., Grossi E., Buckley A.E. (1997). Low folate levels in the cognitive decline of elderly patients and the efficacy of folate as a treatment for improving memory deficits. Arch. Gerontol. Geriatr..

[44] Andreeva V.A., Kesse-Guyot E., Barberger-Gateau P., Fezeu L., Hercberg S., Galan P. (2011). Cognitive function after supplementation with B vitamins and long-chain omega-3 fatty acids: Ancillary findings from the SU.FOL.OM3 randomized trial. Am. J. Clin. Nutr..

[45] Vogiatzoglou A., Refsum H., Johnston C., Smith S.M., Bradley K.M., de Jager C., Budge M.M., Smith A.D. (2008). Vitamin B12 status and rate of brain volume loss in community-dwelling elderly. Neurology.

[46] Hooshmand B., Mangialasche F., Kalpouzos G., Solomon A., Kåreholt I., Smith A.D., Refsum H., Wang R., Mühlmann M., Ertl-Wagner B. (2016). Association of vitamin B12, folate, and sulfur amino acids with brain magnetic resonance imaging measures in older adults: A longitudinal population-based study. JAMA Psychiatry.

[47] Morris M.S., Selhub J., Jacques P.F. (2012). Vitamin B-12 and Folate Status in Relation to Decline in Scores on the Mini-Mental State Examination in the Framingham Heart Study. J. Am. Geriatr. Soc..

[48] Clarke R., Birks J., Nexo E., Ueland P.M., Schneede J., Scott J., Molloy A., Evans J.G. (2007). Low vitamin B-12 status and risk of cognitive decline in older adults. Am. J. Clin. Nutr..

[49] Moorthy D., Peter I., Scott T.M., Parnell L.D., Lai C.-Q., Crott J.W., Ordovás J.M., Selhub J., Griffith J., Rosenberg I.H. (2012). Status of Vitamins B-12 and B-6 but Not of Folate, Homocysteine, and the Methylenetetrahydrofolate Reductase C677T Polymorphism Are Associated with Impaired Cognition and Depression in Adults123. J. Nutr..

[50] McNulty H., Scott J.M. (2008). Intake and status of folate and related B-vitamins: Considerations and challenges in achieving optimal status. Br. J. Nutr..

[51] Willett W. (1998). Nutritional Epidemiology.

[52] ANSES Food Consumption Data from the Individual and National Study on Food Consumption 2 (INCA2). Data.gouv.fr/fr/datasets/donnees-de-consommations-et-habitudes-alimentaires-de-letude-inca-2-3/.

[53] Bates C.J., Prentice A., van der Pols J.C., Walmsley C., Pentieva K.D., Finch S., Smithers G., Clarke P.C. (1998). Estimation of the use of dietary supplements in the National Diet and Nutrition Survey: People aged 65 years and Over. An observed paradox and a recommendation. Eur. J. Clin. Nutr..

